# Fabrication of nanoribbons by dielectrophoresis assisted cold welding of gold nanoparticles on mica substrate

**DOI:** 10.1038/s41598-019-40248-8

**Published:** 2019-03-06

**Authors:** Song-Hyun Cha, Se-Hyeon Kang, You Jeong Lee, Jae-Hyun Kim, Eun-Young Ahn, Youmie Park, Seonho Cho

**Affiliations:** 1 Department of Naval Architecture and Ocean Engineering, Seoul National University, 1 Gwanak-ro, Gwanak-gu, Seoul, 08826 Republic of Korea; 2 College of Pharmacy and Inje Institute of Pharmaceutical Sciences and Research, Inje University, 197 Inje-ro, Gimhae, Gyeongnam 50834 Republic of Korea

## Abstract

Using alternating current electric fields, nanoribbons are fabricated from an aqueous suspension of gold nanoparticles (AuNPs) on mica substrate without resorting to further chemical functionalization of AuNPs. The potential and kinetic energies of AuNPs subjected to attractive forces from a mica substrate provide sufficient energy to pass the diffusion barrier of the gold atoms, which eventually leads to cold welding. A dielectrophoresis force exerted on polarizable particles in a non-uniform electric field contributes to the directed growth of the cold welding that occurs by adjusting the lattice structures of AuNPs. Depending on the concentration of the AuNP suspension, the frequency of the electric field, and the geometry of electrodes, various morphologies of nanoribbons are fabricated. It turns out that the welded region is nearly perfect to provide the same crystal orientation and strength as the rest of the nanostructures, which can be extensively utilized in the fabrication of various nanostructures.

## Introduction

Various nanostructures such as nanoparticles, nanowires, nanoplates, nanoribbons, and so on are attracting increasing attention since they are considered as the main building blocks of nanostructures, which are intended by applying novel bottom-up fabrication methods based on self-assembly^1^. In addition to the self-assembly, an external manipulation with a field has been explored to direct the organization of nanoparticles into arrayed structures^2,3^. Dielectrophoresis (DEP) arises from the polarization of particles in a non-uniform electric field, where the induced dipole moment plays a role of force leading to particle movement^4–6^. The application of alternating current (AC) to suspended particles produces a time-varying dipole, caused by the redistribution of free and bound charges, and assembles micro/nanowires using the mobility and interactions of particles^7–9^. The aggregation of nanoparticles, resulting in ‘*pearl chain formation*,’ has been reported in literature using DEP. The self-assembly approaches seem to lack control over the dimensions, morphology, and electrical properties of the resulting nanostructures^10–12^. Nevertheless, no systematic studies have been done on the interaction among nanoparticles combined with cold welding during the DEP process or the interaction between the aligned nanostructures and substrates. *This paper presents the first electrically controlled growth of thin nanostructures*, *which has immense potential for utilization in the field of nanotechnology*.

Cold-welding is a solid-state welding process that is completed at ambient temperature without noticeable fusion occurring at the welding interfaces^13,14^. As a result, during the welding process, the single-crystalline structures of the welded region were well maintained, barely leaving any defects. Regarding the physics in the cold welding of nanomaterials, it is reported in literature that if supported on compliant elastomers, thin gold films could weld together at remarkably low loads under ambient conditions^15^. It is known that the aggregation of AuNPs occurs during the sample preparation for Atomic Force Microscopy (AFM) measurement, which turns out that the aggregation is originated from a cold welding phenomenon^16^. In the cold-welded nanoparticles, the welding zone has the same lattice structure and is connected to the original particles without any observable grain boundaries. Single-crystalline gold nanowires with diameters between 3 and 10 nm can be cold-welded under the conditions of mechanical contact and low applied pressures^14–17^. For the metallic nanoparticles, however, the cold welding can occur easily on a substrate since there are so-called ‘*self-adjustment*’ that matches crystalline orientations, requiring little external forces. Thus, the idea of cold-welding has become an attractive process as an efficient assembly tool for nanostructures without any loss of original characteristics. Generally, the high quality in the cold welding of suspended AuNPs on mica substrate is attributed to the following ingredients: ‘*oriented-attachment*’ naturally activated in the AuNP suspension on mica substrate, ‘*enhanced atomic diffusion*’ from the high surface to volume ratio of AuNPs, and ‘*surface relaxation and reconstruction*’^16^. No systematic studies, however, have been conducted in combination with cold welding of the AuNPs during the DEP process. However, ready-made AuNPs include a high concentration of stabilizers to prevent the aggregation of AuNPs by any disturbances, which in turn makes the cold-welding difficult. *In this paper*, *through green synthesis*^18^ *followed by the removal of stabilizers*, *cold-welding successfully occurs on mica substrate*, *which can be extensively used for the fabrication tool of elemental nanostructures*. *Without resorting to further chemical functionalization of AuNPs or substrates*, *we use the DEP forces for the directed fabrication of various nanostructures from the AuNPs in liquid suspensions where stabilizers are removed after the green synthesis*. Depending on the concentration of AuNP suspension, the frequency and strength of electric field, and electrode geometry, various nanostructures can be fabricated between the electrodes. It has an immense potential for utilization in the field of nanotechnology. We discuss about DEP-assisted cold welding of AuNPs on mica substrate through the observation of High Resolution - Transmission Electron Microscope (HR-TEM), AFM, and Field Emission - Scattering Electron Microscope (FE-SEM) images. The DEP-assisted cold welding is simulated using LAMMPS^19^ (Large-scale Atomic/Molecular Massively Parallel Simulator) molecular dynamics (MD) computations.

## Results and Discussions

### Cold welding of AuNPs

**Figure 1:**
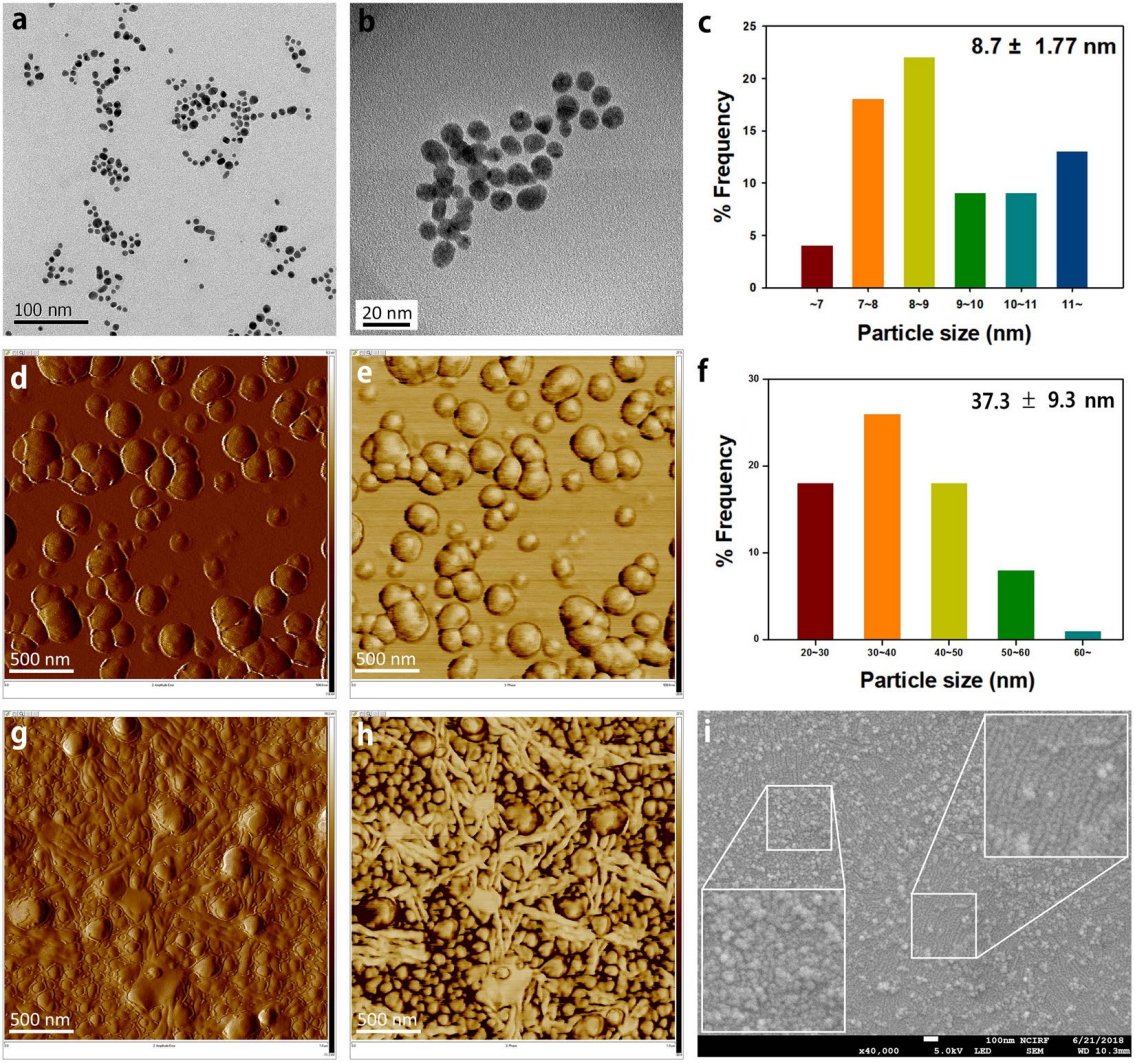
Characterization of morphology for synthesized AuNPs: All AuNPs in the images were synthesized with HAuCl_4_•3H_2_O as a precursor ion. The reducing agent was a green tea extract. Green synthesis: 75 AuNPs from a randomly selected area in a HR-TEM image (**a**) were used to measure the average diameter. Size histogram in (**c**) shows an average size of 8.7 ± 1.77 nm. Cold welding on mica substrate: 75 AuNPs from a randomly selected area in amplitude error image in (**d**) were used to measure the average diameter. Size histogram in (F) shows an average size of 37.3 ± 9.3 nm. DEP-assisted cold welding: AC electric potential of 1 V and the frequency of 100 Hz were used in the amplitude error (**g**) and the phase (**h**) images of AuNPs. In the FE-SEM image (**i**) nanoribbons grew between AuNPs where the concentration of AuNPs was appropriate (See right upper inset). If the concentration was high, the AuNPs simply grew their diameters (See left lower inset).

Figure 1(a,b) show the HR-TEM images of AuNPs synthesized using green tea extract as a reducing agent (Green synthesis^18^). We noticed that the size of the AuNPs measured by the HR-TEM images was smaller than 20 *nm* as shown in Fig. 1(b,c).

When measuring the size of the AuNPs, we observed a significant difference; the size of the AuNPs in the AFM image (20~40 *nm*) is much larger than those in the HR-TEM image (~20 *nm*) as shown in Fig. 1(c,f), due to the cold welding phenomenon. It is well known that there is a strong attractive force between Au atoms and silicate in mica substrate^15^. Due to the attractive forces from the mica substrate, sphere-shaped AuNPs grow only on the plane parallel to the mica substrate. Compared to nanowires and nanofilms, the AuNPs in the suspension are able to freely rotate so that the cold welding goes along to adjust the lattice structure of the AuNPs.

### DEP-assisted cold welding

**Figure 2:**
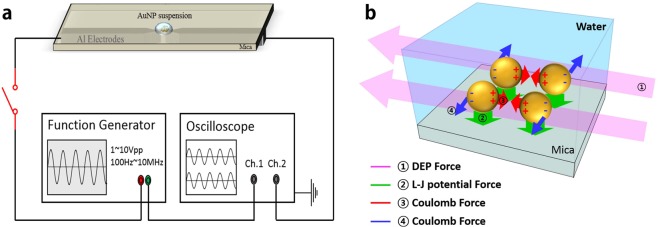
Schematics of DEP device (**a**) and the mechanism of exerting force (**b**).

If the aqueous suspension of AuNPs on mica substrate is subjected to an electric field, the cold welding with DEP forces lead to various shapes of nanostructures as shown in Fig. 1(g,h). The phase image (h) confirms that the grown structures were made of gold. In the FE-SEM image (i), a nanoribbon grew between the AuNPs where the concentration of the AuNPs was appropriate (See right upper inset in (i)). For the DEP, a function generator is operated at a frequency between 100 Hz and 10 MHz and a peak-to-peak voltage *V*_pp_ of 1–10 *V*, as shown in Fig. 2(a).

Nanoribbon chaining occurs such that AC field generates a DEP force exerted on the AuNPs adjacent to the electrodes and a dipole–dipole interaction between AuNPs far from the electrodes. The suspended AuNPs on mica substrate in an electric field are subjected to the following forces; ① *F*_*DEP*_ (DEP force), ② *F*_*mica*_ (Lennard-Jones potential force from mica substrate), ③ *F*_*Coulomb*_ (Coulomb force between charged atoms), and *F*_*Lorentz*_ (Lorentz force in electric field) in Fig. 2(b). The DEP force^4^, ${F}_{DEP}=2\pi {\varepsilon }_{m}\mathrm{Re}[K(\omega )]{a}^{3}\nabla {E}_{rms}^{2},$ is proportional to particle volume *a*^3^ and the gradient of electric field squared $\nabla {E}_{rms}^{2}$. *ε*_*m*_, *E*_*rms*_, and *ω* are the permittivity of the suspending medium, the root-mean-squared value and angular frequency of electrical field, respectively. Re[*K*(*ω*)] is the real part of the Clausius–Mossotti factor. The DEP force does not contribute to the aggregation of AuNPs but accumulates the AuNPs near the tip of the nanostructure to sustain its growth. *F*_*Lotentz*_ is eliminated since the overall charge of each AuNP equals zero. *F*_*Coulomb*_ attracts AuNPs for chaining and *F*_*mica*_ induces the cold welding, after which the grown AuNPs are subjected to a repulsive force ④ *F*_*Coulomb*_ perpendicular to the DEP force (Refer to Supplement: Movie 1).

**Figure 3:**
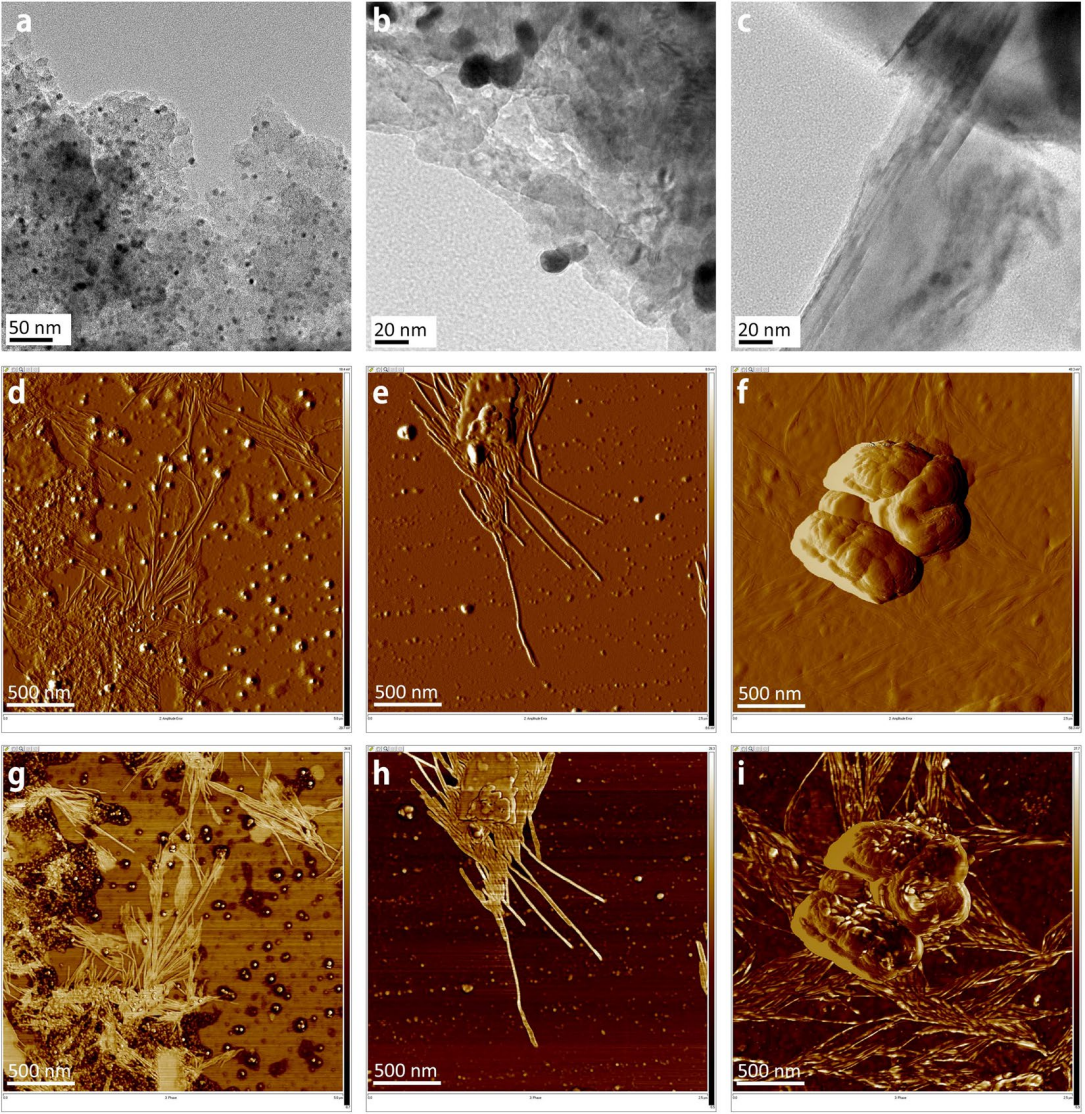
DEP-assisted cold welding of AuNPs on mica substrate. HR-TEM images: The dark spots indicate AuNPs and the blurred stains show a cloud of assembled nanoribbons around the AuNPs. AFM amplitude error images: The nanoribbons started to grow between the aggregated AuNPs (**d**), around the aggregated AuNP (**f**) on mica substrate. The growth continues using the AuNPs around the tip of nanoribbon until no more AuNPs are available (**e**). AFM phase image: Very thin nanoribbons around a big cluster of AuNPs were barely recognizable in amplitude error image (**f**) but found in phase image (**i**).

In the HR-TEM images of Fig. 3(a–c), the sample was obtained by placing a HR-TEM grid on mica substrate subjected to AC electric field. The frequency and strength of AC electric field were 100 kHz and 5 V, respectively. The electric field strength and AuNP concentration are of major importance because they must exceed a threshold value for the nanoribbons to start growing. Since the gap of the electrodes is larger than 100 *μm*, the aggregated AuNPs play the role of conductive objects as shown in (d).

### Parametric study

**Figure 4:**
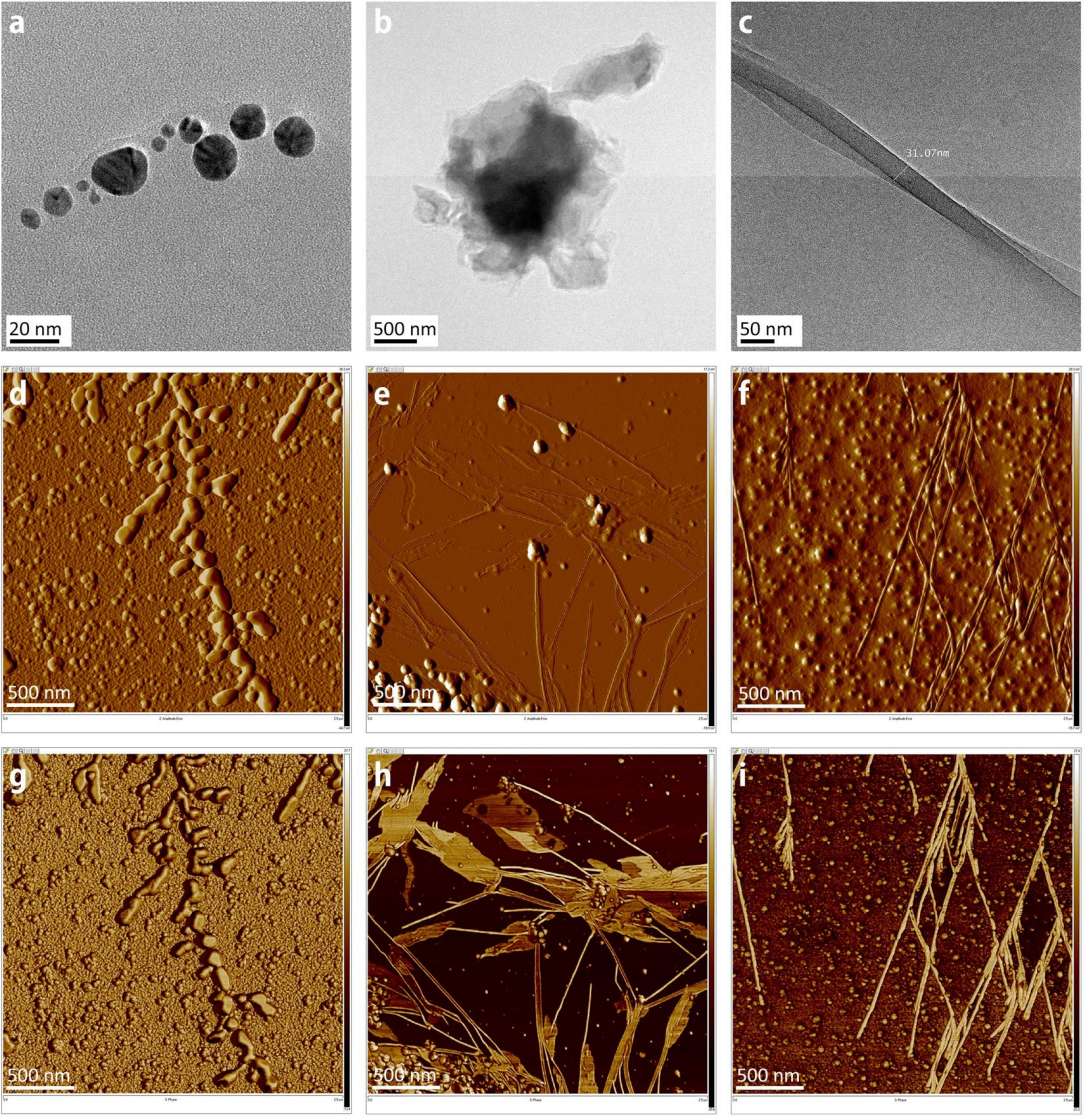
Effect of AuNP concentration. HR-TEM images: AuNPs synthesized using green tea extract as a reducing agent (**a**) and pipetted on mica substrate (**b**). The width and height of fabricated nanoribbon (**c**) were 31.07 *nm* and 5 *nm*, respectively. AFM amplitude error and phase images: With the original solution, a frequency of 1 MHz, a peak-to-peak voltage *V*_*pp*_ of 1 V, the AuNPs were aligned but failed to form continuous nanoribbons (**d**,**g**). With a twice-diluted solution, a frequency of 100 kHz, and a maximum voltage of 5 V, nanoribbons or nanoleaves are formed (**e**,**h**). When the solution is 5-times diluted at a frequency of 100 kHz and a maximum voltage of 5 V, no more nanoleaves are formed (**f**,**i**).

When subjected to an electric field, an AuNP has polarity due to the distribution of induced charge. The dipole–dipole attraction of AuNPs does not occur when the concentration of the AuNPs is too low. If the concentration of the AuNPs is too high, the AuNPs simply grow in a radial direction rather than forming nanoribbons as shown in Fig. 4(d,g).

The exerted DEP force makes the grown AuNPs gather but fails to form a continuous nanoribbon due to the large size of the AuNPs. The ‘*pearl-chain formation*’ is associated with the induced dipole-dipole interaction between AuNPs that are brought together by the DEP forces. With a twice-diluted suspension, a long nanoribbon is grown between the aggregated AuNPs in (e, h). The AuNPs gather on the surface of the electrode where a big gradient of electric field is observed, which induces the growth of a nanoribbon and then the increase of the gradient of electric field as well. The gradient of electric field enforces the AuNPs to move in the direction of the electric field. This DEP transport increases the density of the AuNPs and eventually induces a polarity-polarity attraction between AuNPs. Chaining of AuNPs is conducted by the Lorentz and Coulombic forces from the distribution of surface charge in the AuNPs. The threshold effect of the AuNP concentration implies that aggregation occurs only when the concentration of AuNPs accumulated near the tip of nanoribbon is sufficiently high to sustain the growth of nanoribbon and prevent depletion effects at the tips. In the phase image (h), we observe thin nanoleaves forming from ultra-small AuNPs, where the highest field gradients are required and found near the conductive objects. Due to Coulomb forces, if the neighboring nanoribbons are close enough, polarized nanoribbons attract the neighboring ones to form strands parallel to their long axes. A nanoleaf is formed from the cold welding of nanoribbon strands. For a 5-times diluted suspension, no more nanoleaves are found as shown in (f, i).

### Effect of frequency

**Figure 5:**
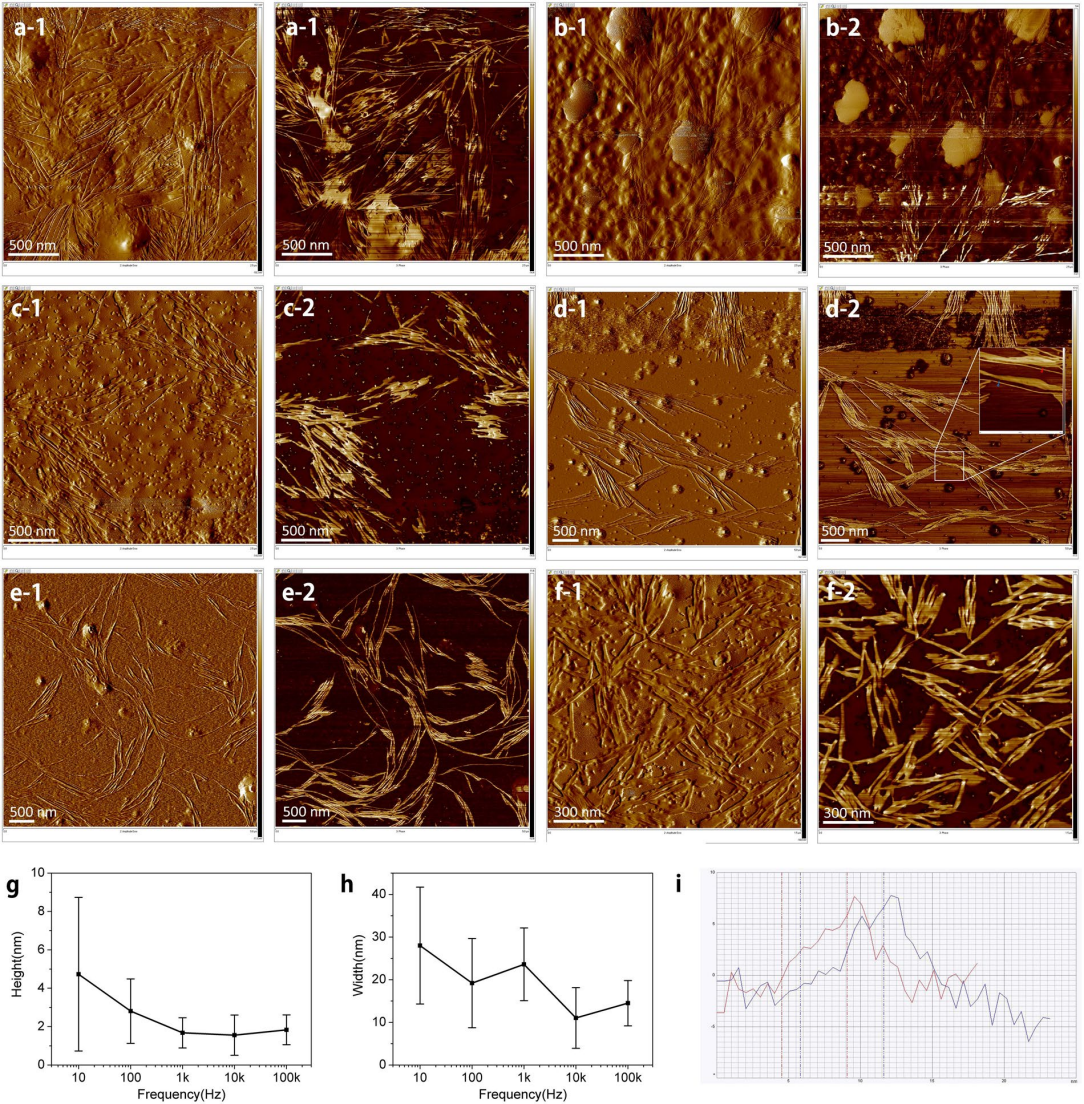
Effect of frequency The frequencies of exerted electric field are 100 Hz (**a**); 1 kHz (**b**); 10 kHz (**c**); 100 KHz (**d**); 1 MHz (**e**); 10 MHz (**f**). By varying the frequency, we obtain the distribution of height (**g**) and width. (**h**) Section analysis (**i**) was performed to obtain the dimensions for the case of 100 kHz. (Refer to the red and blue spots in the inset of d-2).

We investigate the characteristics of nanoribbons by varying the frequency of exerted electric field. In Fig. 5, most of the nanoribbons are generally aligned perpendicular to the electrodes while slightly deviated ones are present due to conductive objects. Further increase of frequency yields nanoribbons with no directivity in Fig. 5(e,f). By increasing the frequency, the long, thick, and winding nanoribbons are turned into short, thin, and straight ones in Fig. 5. Depending on the size of the synthesized AuNPs, 1.6~4.7 nm high (g), 11~28 nm wide (h), and 0.4~1 μm long nanoribbons are constructed.

### Effect of electrode geometry

The strength of the electric field does not significantly affect the aggregation but contributes in the aligning of the AuNPs. Thus, a small electric potential of 1 *V*_*pp*_ is applied to prevent possible thermal effects. Also, the AuNP suspension was pipetted onto the top of the mica substrate whereas the electrodes were attached on the bottom. The direction of nanoribbon growth is controlled by disturbing the homogeneity of electric field, which is naturally done by forming conductive objects in the gap between the electrodes. Such objects create a gradient in the electric field and cause the nanoribbon to grow toward them. For the controlled applications of DEP, a non-uniform electric field established by electrode geometry is necessary.

**Figure 6:**
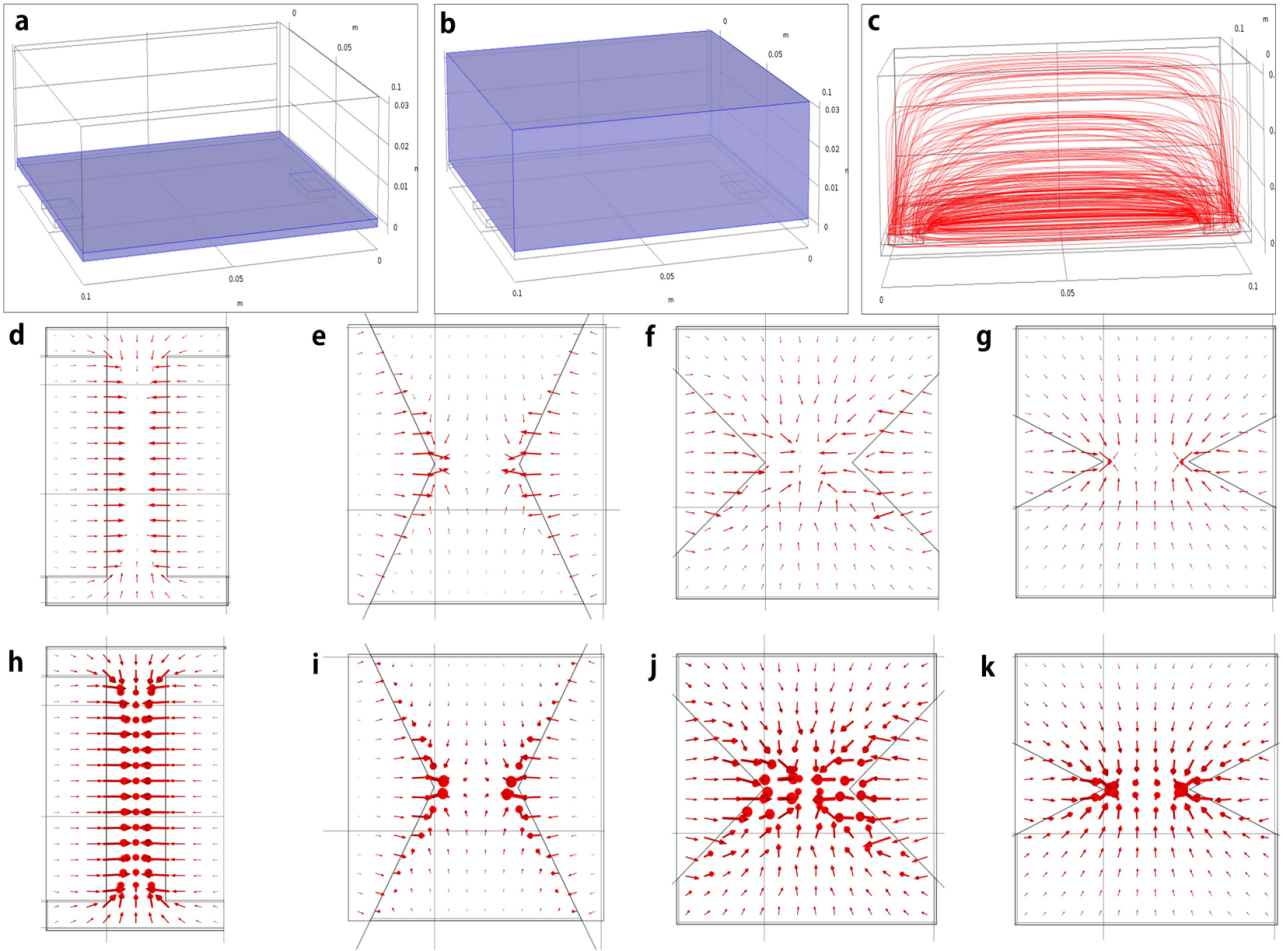
Effect of electrode shape on electric field strength. Electrostatic analysis: The mica has dimensions of 0.1 m × 0.1 m (**a**: mica substrate) and the water 0.1 m×0.1 m×0.03 m (**b**: water). The electrodes are placed on the bottom surface of mica and the gap between electrodes is 0.080 mm. When the peak to peak voltage of *V*_*pp*_ = 10 is applied, the streamline of electric field vector is shown in (**c**). Electric field for various electrode shapes: The red arrows represent the magnitude and direction of electric field in the space of water. The effect of electrode geometry on the electric field is investigated for the electrode angles of parallel 0° (**d**), 30° (**e**), 45° (**f**), 60° (**g**), in 2D; parallel 0° (**h**), 30° (**i**), 45° (**j**), 60° (**k**) in 3D.

Since the DEP force is proportional to the gradient of electric field squared, we investigate the effect of electrode geometry on $\nabla {E}_{rms}^{2}$. To obtain a qualitative distribution of $\nabla {E}_{rms}^{2},$ mica, electrodes, and water are modeled by a multi-physics analysis code, COMSOL. Figure 6 shows the mathematical models of the mica (a) and the water (b). The electrodes are attached on the bottom surface of mica to avoid direct contact with water. Figure 6(c) shows the streamline of the obtained electric field vector when the peak to peak voltage of *V*_*pp*_ = 10 is applied.

**Figure 7:**
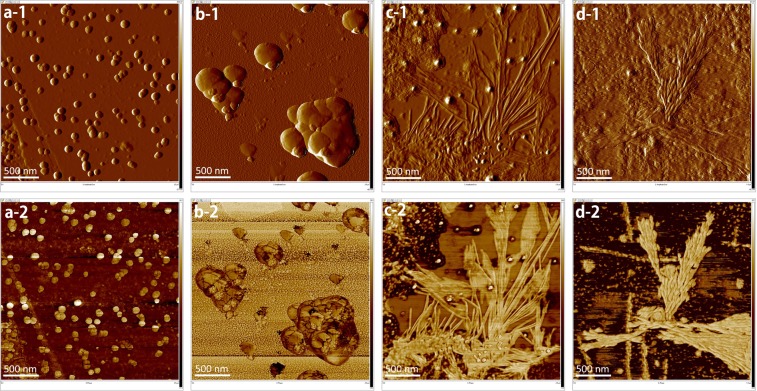
Effect of electrode geometry: For a 10-times diluted suspension at a frequency 100 kHz and exerted electric potential of 5 V_pp_, AFM amplitude error and phase images are shown for the cases of parallel electrode (**a**,**b**) and arrowhead electrode (**c**,**d**).

Electrostatic analysis is carried out to investigate the effect of electrode geometry on the electric field in the space of water. In 2-dimensional cases, the parallel electrodes produce no electric field along the vertical centerline of electrode gap in Fig. 6(d). By varying the inclined angle of the electrode to the vertical centerline, various electric fields are observed in Fig. 6(e) for 30°, (f) for 45°, and (g) for 60°. At the midpoint between the tips of the electrodes, the 45° inclined electrodes produce the strongest electric field. For the 3-dimensional cases, top views are shown in Fig. 6(h–k). The distributions of the electric field are similar to the 2-dimensional cases except the addition of a downward (from top to bottom) electric field. At the midpoint, the 45° inclined electrodes in Fig. 6(j) also produce the strongest electric field with more complicated distribution due to the downward one. For the parallel case, the electric field disappears along the centerline and thus only cold welding occurs due to the weak DEP force as shown in Fig. 7(a,b). Among the inclined cases, the 45° inclined one showed the best performance and produced many nanostructures due to sufficient DEP force in (c) and (d).

### MD Simulation of DEP-assisted cold welding

**Figure 8:**
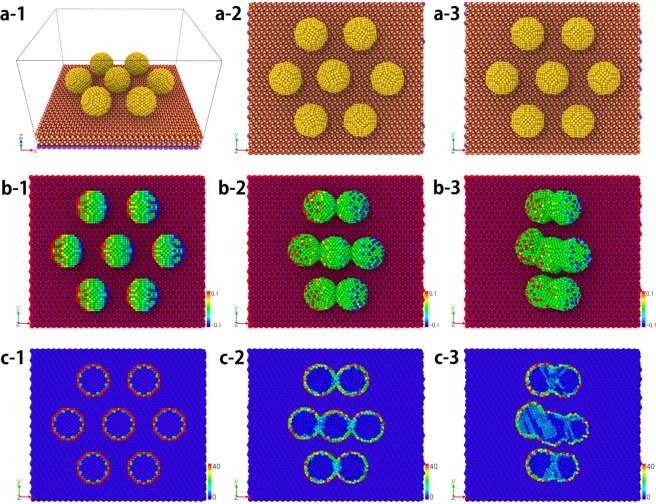
MD simulation of seven AuNPs on mica substrate: The columns show the results at the time of 0, 40, and 80 (ps), respectively. (**a**) Without electric field: AuNPs are not aggregated since they are located far away. (**b**) With electric field: AuNPs are aligned in the direction of the electric field (Refer to Supplement: Movie 1). (**c**) Centro-symmetry parameter (Refer to Supplement: Movie 2).

The MD method and the numerical model for the mica substrate are constructed, based on ref.^16^. We consider the electrical interactions as well as the van der Waals force, which is a short range force between the atoms in Au and mica. However, the electrostatic force, a long range force, is induced by charges due to electric field and can attract atoms located far away. Induced charge distribution is considered on the surface of the AuNPs. Since the charged surface atoms are subjected to additional forces like Lorentz and Coulomb forces, the motion of AuNPs is affected by an external electric field (Refer to Supplement: Movie 1). To observe the phenomenon that AuNPs are affected by the external electric field, we performed LAMMPS^19^ simulation for 7 AuNPs as shown in Fig. 8a. Each AuNP is composed of 959 Au atoms and all AuNPs are attached on Muscovite mica substrate. The total number of atoms is 49,721. The interatomic potential employed to consider interaction between Au-Au atoms in the simulations is described by the embedded atom method (EAM)^20^ where the potential is described by a pair potential and a function of the electronic density. Simulations are performed such that all atoms maintain an isothermal state of 358 K under x-directional electric field, $E=0.05\,(V/\dot{{\rm{A}}})$. The contour in an AuNP indicates the magnitude of charge.

Without an electric field, the particles are not aggregated (a-2, a-3) since they are located far away. Figure 8(b) shows the result of simulation, where the AuNPs are aligned in the direction of the electric field. The atoms parallel to the direction of the electric field are charged with opposite polarity and attract each other (b-2). However, the aggregated particles perpendicular to the electric field direction are charged with same polarity, which results in a repulsive force (b-3). The AuNPs grow in the plane parallel to the mica substrate, maintaining regular lattice structures. The welded AuNPs retain their crystalline fcc structure even in the welded region, in agreement with the known experimental findings^14^ that the few defects introduced in the process reconstruct to restore the fcc structure. In the cold welding process, the AuNPs go through an iterative process of clustering and migrating until they are sufficiently stabilized.

**Figure 9:**
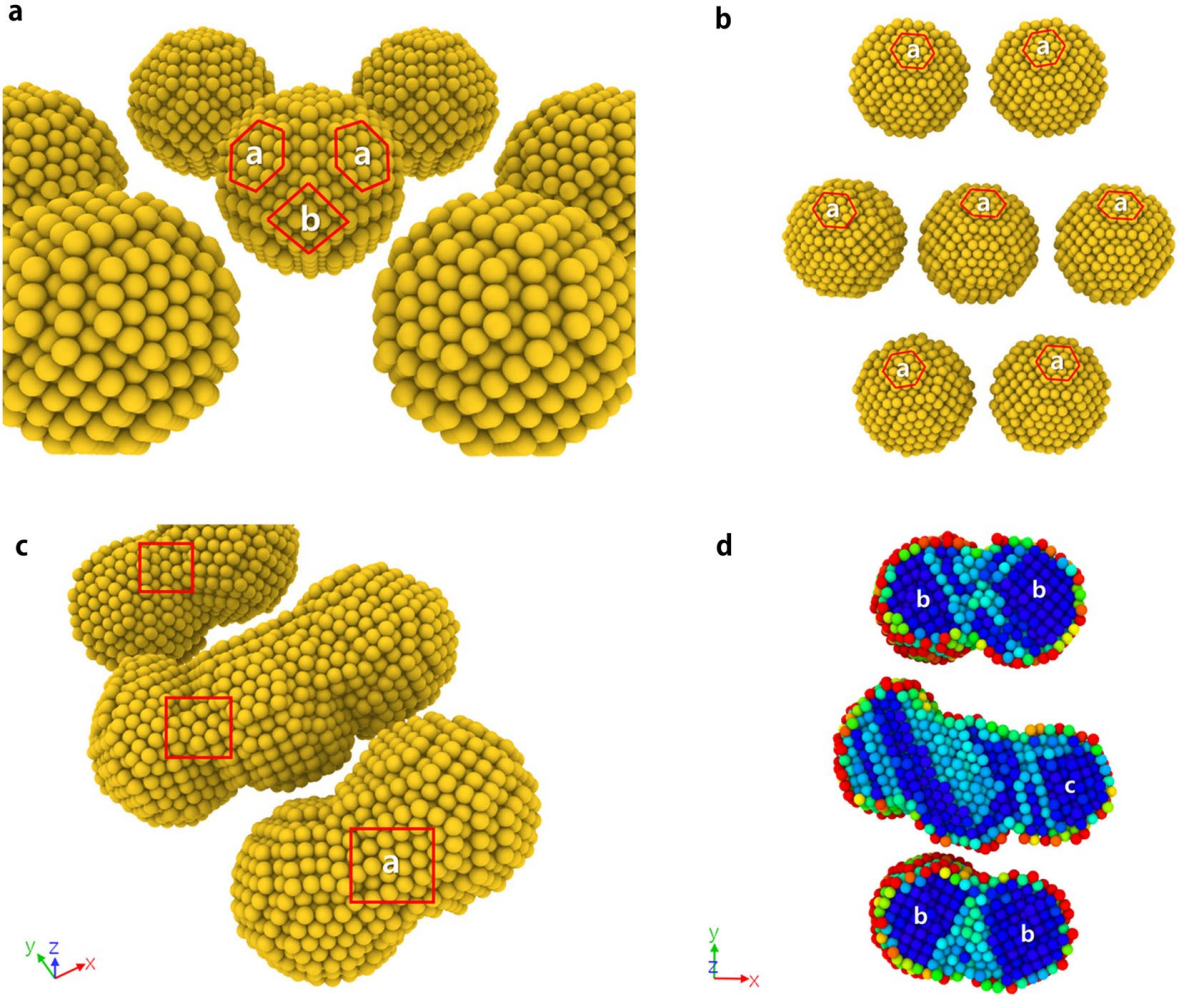
Lattice structure of seven AuNPs on mica substrate: ‘a’, ‘b’, and ‘c’ surfaces in each figure represent the {111}, {100} and {211} planes of fcc structure, respectively. (**a**) Initial state: The surface of AuNP consists of {111} and {100} planes. (**b**) After 35 ps: The AuNPs are about to be cold-welded. (**c**) After 80 ps: {111} planes are found at interfaces of cold welded AuNPs^25^. (**d**) Centro-symmetry parameter after 80 ps.

To measure the quality of lattice structures, we consider the history of centro-symmetry parameter during the cold welding. The centro-symmetry parameter^21^ in Fig. 8(c) is used to identify defects in crystals such as stacking faults in fcc (face-centered cubic) structures. The stacking faults expand from the welding surface as cold welding progresses. The contour in an AuNP indicates the magnitude of stacking faults. After some relaxation period, the regular fcc structure is quickly recovered (Refer to Supplement: Movie 2). The centro-symmetry analysis shows that the cold welding occurred with low stress, and at the end of the process, a crystalline structure with very few defects was achieved, recovering most of original characteristics of the pristine AuNPs. Figure 9 shows the surface of an AuNP consisting of {111} and {100} planes.

The AuNPs rotate in the same direction since the unbalance forces from mica substrate are much bigger that those from Coulomb forces arising from the lattice mismatch of two AuNPs. Just before the cold welding, all the AuNPs are aligned so that {111} plane of each AuNP is parallel to that of the others in Fig. 9(b). After the cold welding shown in Fig. 9(d), {100} planes are found in the z-directional section of top and bottom AuNPs. In the middle AuNP, {211} planes are found everywhere except for the stacking fault region as shown in the contour of centro-symmetry parameter.

In summary, the DEP-assisted cold welding forms straight nanoribbons adjusting lattice structures. The cold-welding of AuNPs generally occurs in a radial and in-plane direction by the interactions with mica substrate. In the presence of DEP forces, however, cold-welding is directed to the direction of electric field. It turns out that the welded region is nearly perfect to provide the same crystal orientation and strength as the rest of nanostructures, which can be extensively utilized in the fabrication of various nanostructures.

## Methods

### Experiment

(*Preparation of green tea extract*) Dried green tea leaves used in the present study were a donation from the Institute of Hadong Green Tea (Hadong, Gyeongnam, Republic of Korea). The dried leaves were pulverized by using a blender and deionized water (2 L) was added to the pulverized leaves (200 g). Sonication was conducted at ambient temperature for 1 h to extract materials. The extraction process was repeated three times. Water fraction containing extracted materials was pooled and filtered using Whatman filter papers. Centrifugation was performed (3,000 g force, 18 °C, 25 min) and the supernatant was pooled. The pooled supernatant was subjected to syringe-filter and the filtered solution was freeze-dried. The freeze-dried powder was dissolved in de-ionized water to make a final concentration of 2% (w/v). This solution was used as a stock solution and utilized for the green synthesis.

(*Green synthesis using green tea extract*) For the green synthesis^18^ of AuNPs, gold (III) chloride trihydrate (HAuCl_4_·3H_2_O) was purchased from Sigma-Aldrich (St. Louis, MO, USA). The mixture of the extract (final concentration of 0.03%), hydrochloroauric acid trihydrate (final concentration of 0.5 mM) and sodium hydroxide (final concentration of 1 mM) was prepared in deionized water (final volume 2 mL). Then the mixture was incubated in a 80 °C dry oven for 2 *h*. The mixture was subjected to centrifugation (14,000 g force, 20 °C, 20 *min*). The supernatant was removed and the pellet was re-dispersed with the same volume of deionized water.

(*DEP on mica substrate*) The synthesized AuNP suspension was loaded onto the following substrates and dried at ambient temperature overnight.

**Figure 10:**
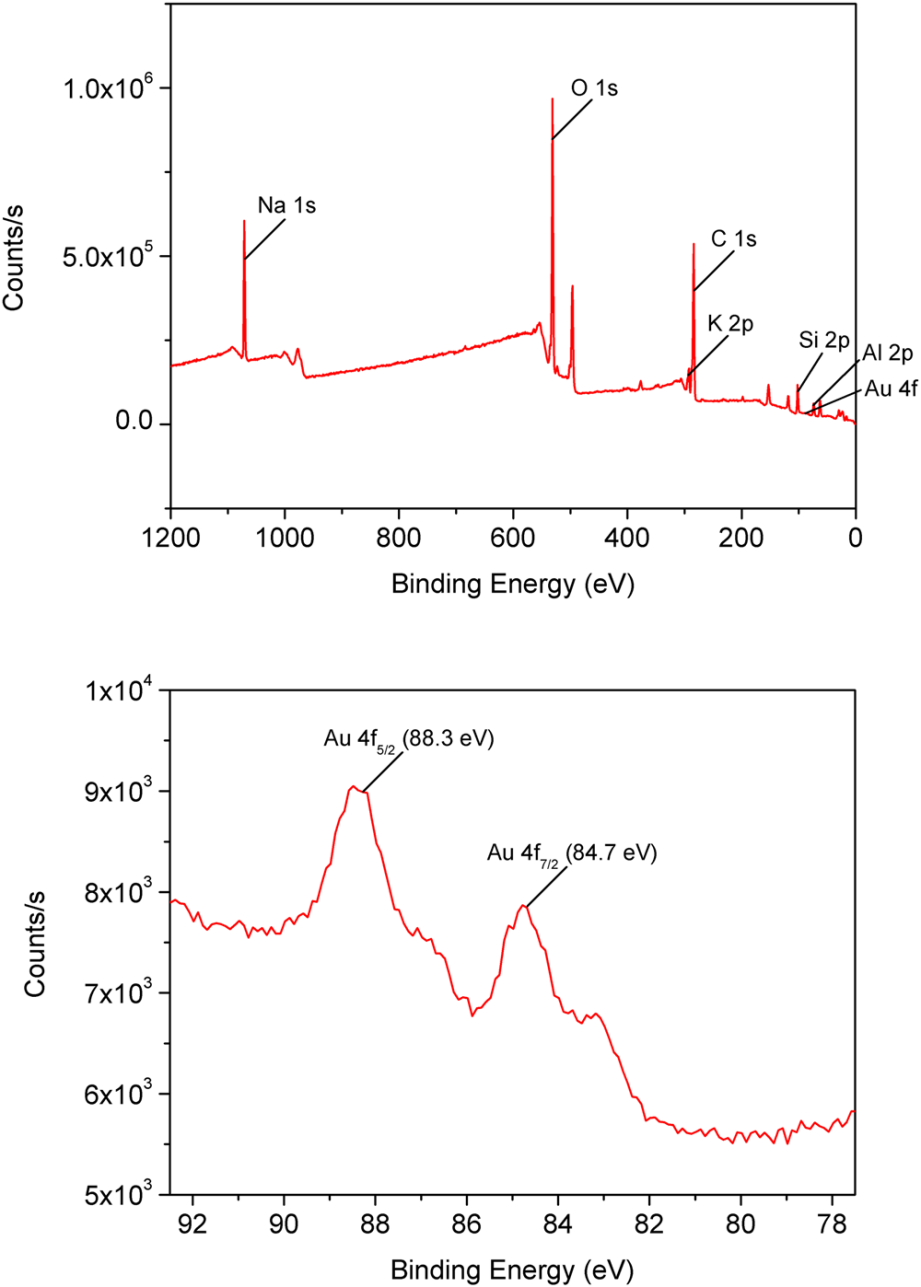
XPS scan spectra of nanoribbon on mica substrate. X-ray Photoelectron Spectroscopy (XPS) was performed on nanoribbons which was loaded onto a mica substrate. XPS spectra confirmed that the nanoribbon AuNPs are made of Au(0). The spectra clearly showed the doublet photoelectron peaks of Au 4 f. The binding energy was observed as 84.7 eV for Au 4f_7/2_ and 88.3 eV for Au 4f_5/2_ in the lower figure.

- TEM grid (a carbon-coated copper grid; carbon type-B, 300 mesh copper grid, Ted Pella, Inc., Redding, CA, USA). The AuNP suspension was pipetted onto the TEM grid.
- Mica substrate (grade V-1, 25 mm × 25 mm length, 0.15 mm thick, SPI Supplies Division of Structure Probe, West Chester, PA, USA)
- Mica substrate with electric field applied. The electrodes were made of aluminum and attached on the bottom of mica. The non-uniform fields were created using arrowhead electrodes whose gap size was 100 μm. The AuNP suspension was pipetted onto the top of the mica to avoid direct contact with the electrodes. A 1~20 V peak-to-peak sinusoidal signal of frequency between 100 Hz and 10 MHz was applied to the electrodes (SIGLENT function generator; Dual-channel, with bandwidth up to 60 MHz, and amplitude up to 20 V_*pp*_.)
- To characterize the nanostructures using spectrum techniques, K-Alpha X-ray Photoelectron Spectrometer (XPS) System was performed with a monochromatic Al Kα radiation of energy 1,486.6 eV (Thermo Fisher Scientific, UK). X-ray energy used was 12 kV and 72 W.
- The major peaks were originated from the mica and the remaining reducing agent. The peaks for C 1 s, N 1 s, O 1 s, Si 2p, and Al 2p were from the mica substrate. A small peak of Au 4 f was observed. As shown in Fig. 10, doublet photoelectron peaks of Au 4 f were composed of Au 4f_7/2_ at 84.7 eV and Au 4f_5/2_ at 88.3 eV. These peaks can be assigned to Au(0) of nanoribbons.

(*Characterization using TEM*, *AFM*, *and FE-SEM*) A JEM-3010 microscope operated at 300 kV (JEOL Ltd., Tokyo, Japan) was utilized to obtain HR-TEM images. The morphology of the nanostructures was characterized by AFM (Dimension^®^ Icon™ operated in tapping mode), and by FE-SEM (JSM-6700F; JEOL Ltd., Tokyo, Japan).

### MD simulation

To verify the effects of the electric field on the AuNPs on mica substrate, MD simulations are performed using the LAMMPS code with time steps of 0.5 fs. The MD numerical model for mica substrate is constructed, based on ref.^16^.

(*Application of forces*) On the surface of the AuNPs, the induced charge distribution is considered. Since the charged surface atoms are subjected to additional forces like Lorentz and Coulomb forces, the motion of AuNPs is affected by the external electric field. The DEP force for AuNPs can be computed as ${F}_{DEP}=2\pi {\varepsilon }_{m}\mathrm{Re}[K(\omega )]{a}^{3}\nabla {E}_{rms}^{2}$. If the gradient of the electric field is small, all the AuNPs in the electric field are subjected to homogeneous DEP forces which do not contribute to the aggregation of AuNPs. Also, when it comes to MD simulations, the interaction between the atoms in the AuNPs should be taken into account. The computation procedure of Coulomb forces due to the charge of AuNPs induced from the given electric field has been embedded in the LAMMPS code (Refer to *Supplement: Charge distribution method* for details). A charge distribution method is developed, based on the electrostatic shielding that the electric field disappears at the internal atoms of a metal nanoparticle subjected to an electric field. The first step is to identify the surface atoms in the AuNP, which is easily carried out by examining the magnitude of interaction potential energy in each atom of the AuNP since the metal nanoparticle maintains the lattice structure and the interaction potential energy tends to increase at surface atoms. Free electron distribution in an AuNP induces the charge distribution, which can be obtained by solving a linear system that enforces the overall electric field disappears inside the AuNP. The atoms in the AuNP are subjected to the following forces; ${F}_{Au}={F}_{EAM}+{F}_{mica}+{F}_{Lorentz}+{F}_{Coulumb},$ where *F*_*EAM*_ is from surrounding Au atoms, *F*_*mica*_ is from mica substrate, Lorentz force *F*_*Lotentz*_ in electric field, and *F*_*Coulomb*_ between charged atoms. *F*_*Lotentz*_ is eliminated since the overall charge of each AuNP is equal to zero and *F*_*EAM*_ has little effect due to the long distance between the AuNPs. Thus, the interactions between AuNP-AuNP occur only by the *F*_*mica*_ and *F*_*Coulomb*_.

(*MD Simulation*) Seven AuNPs of fcc lattice structure (lattice constant $4.078\,\dot{{\rm{A}}}$) are modeled to be attached to the mica substrate. The AuNPs are attached to the surface of mica substrate by the exchange mechanism of potassium metal ion and the AuNPs. The necessary potential parameters for the mica substrate are obtained from refs^22–24^. Periodic boundary conditions are imposed in the x and y directions. We construct a unit cell of mica substrate that can be extensively used according to the size and the number of AuNPs. The MD simulations are performed under the condition that atoms within 1 nm from the bottom are fixed and the others are free to move. An energy minimization of the MD system is conducted using a steepest descent algorithm. Furthermore, to remove the instability of the system, an isothermal (NVT) simulation is conducted for 100 ps at 300 K with a time step of 0.5 fs. After thermal equilibrium, an electric field of $0.05({\rm{V}}/\dot{{\rm{A}}})$ is applied to the lateral direction. Since the sample of AuNP suspension is dried on mica at an ambient temperature in real experiments, a Nose-Hoover thermostat is introduced to keep the temperature at 300 K in a canonical ensemble.

## Supplementary information

Supplementary information

motion of seven AuNPs on mica substrate

Evolution of centro-symmetry parameters for seven AuNPs

## Data Availability

All data are available in the manuscript or the Supplementary Materials.

## References

[1] Xia Y (2003). One-Dimensional Nanostructures - Synthesis, Characterization, and Applications. Adv. Mater..

[2] Garcia-Sanchez P, Arcenegui JJ, Morgan H, Ramos A (2015). Self-assembly of metal nanowires induced by alternating current electric fields. Applied Physics Letters.

[3] Kim SJ, Jang DJ (2005). Laser-induced nanowelding of gold nanoparticles. Appl. Phys. Lett..

[4] Xiong X (2007). Directed assembly of gold nanoparticle nanowires and networks for nano-devices. Appl. Phys. Lett..

[5] Ranjan N, Mertig M, Cuniberti G, Pompe W (2010). Dielectrophoretic Growth of Metallic Nanowires and Microwires: Theory and Experiments. Langmuir.

[6] Dong L (2005). Dielectrophoretically controlled fabrication of single-crystal nickel silicide nanowire interconnects. Nano Lett..

[7] Ranjan N, Vinzelberg H, Mertig M (2006). Growing one-dimensional metallic nanowires by dielectrophoresis. Small.

[8] Hermanson KD (2001). Dielectrophoretic assembly of electrically functional microwires from nanoparticle suspensions. Science.

[9] Bhatt KH, Velev OD (2004). Control and Modeling of the dielectrophoresis assembly of on-chip nanoparticle wires. Langmuir.

[10] Kretscher R, Fritzsche W (2004). Dielectrophoretic Pearl Chain Formation of Nanoparticles in Microelectrode Gaps by Dielectrtophoresis. Langmuir.

[11] Gierhart BC, Howitt DG, Chen SJ, Smith RL, Collins SC (2007). Frequency Dependence of Gold Nanoparticle Superassembly by Dielectrophoresis. Langmuir.

[12] Sam M, Moghimian N, Bhiladvala RB (2016). Field-directed chaining of nanowires: towards transparent electrodes. Materials letters.

[13] Wagle DV, Baker GA (2015). Cold welding: a phenomenon for spontaneous self-healing and shape genesis at the nanoscale. Mater. Horiz..

[14] Lu Y, Huang JY, Wang C, Sun S, Lou J (2010). Cold welding of ultrathin gold nanowires. Nat. Nanotechnol..

[15] Ferguson GS, Chaudhury MK, Sigal GB, Whitesides GM (1991). Contact adhesion of thin gold films on elastomeric supports: cold welding under ambient conditions. Science.

[16] Cha S-H (2016). Cold welding of gold nanoparticles on mica substrate: self-adjustment and enhanced diffusion. Sci. Rep..

[17] Pereira ZS, da Silva EZ (2011). Cold welding of gold and silver nanowire: a molecular dynamics study. J. Phys. Chem. C.

[18] Park Y (2011). Polysaccharides and phytochemicals: a natural reservoir for the green synthesis of gold and silver nanoparticles. IET Nanobiotechnol..

[19] Plimpton S (1995). Fast parallel algorithms for short-range molecular dynamics. J. Comput. Phys..

[20] Foiles SM, Baskes MI, Daw MS (1986). Embedded-Atom-Method functions for the FCC metals Cu, Ag, Au, Ni, Pd, Pt, and their alloys. Phys. Rev. B.

[21] Kelchner CL, Plimpton SJ, Hamilton JC (1998). Dislocation nucleation and defect structure during surface indentation. Phys. Rev. B.

[22] Heinz H, Koerner H, Anderson KL, Vaia RA, Farmer BL (2005). Force field for mica-type silicates and dynamics of octadecylammonium chains grafted to montmorillonite. Chem. Mater..

[23] Srivastava R, Singh JK, Cummings PT (2012). Effect of electric field on water confined in graphite and mica pores. J. Phys. Chem. C.

[24] Richardson SM (1982). Crystal structure of a pink muscovite from Archer’s Post, Kenya: Implications for reverse pleochroism in dioctahedral micas. Am. Mineral..

[25] Grouchko M (2014). Merging of metal nanoparticles driven by selective wettability of silver nanostructures. Nat. Commun..

