# Supplementary material for: Fabrication of nanoribbons by dielectrophoresis assisted cold welding of gold nanoparticles on mica substrate
Source: Sci Rep. 2019 Mar 6;9:3629. doi: 10.1038/s41598-019-40248-8 (PMC6403349; doi:10.1038/s41598-019-40248-8)
Supplement: Supplementary file 1 — Supplementary information [file 41598_2019_40248_MOESM1_ESM.docx]

**Supplementary information**

**TITLE:** Fabrication of nanoribbons by dielectrophoresis assisted cold welding of gold nanoparticles on mica substrate

**AUTHOR NAMES:** Song-Hyun Cha^1^, Se-Hyeon Kang^1^, You-Jeong Lee^2^, Jae-Hyun Kim^1^, Eun-Young Ahn^2^, Youmie Park^2*^, Seonho Cho^1*^

**AUTHOR ADDRESS:**

^1^Department of Naval Architecture and Ocean Engineering, Seoul National University,
1 Gwanak-ro, Gwanak-gu, Seoul 08826, Republic of Korea

^2^College of Pharmacy and Inje Institute of Pharmaceutical Sciences and Research,
Inje University, 197 Inje-ro, Gimhae, Gyeongnam 50834, Republic of Korea

^*^Co-corresponding authors’ E-mail address: secho@snu.ac.kr, youmiep@inje.ac.kr

***1. Charge distribution method***

Let **x***_i_* and **x***_j_* be the positions of inner atom *i* and surface atom *j*, respectively. The objective is to determine the charge *q_j_* of surface atoms when an external electric field is given. Since the electric field vector vanished at inner atoms, the following system of linear equations should be satisfied.

$\mathbf{E}(\mathbf{x}_{i})=\mathbf{E}_{external}(\mathbf{x}_{i})+\sum_{j=1}^{surface} q_{j}\frac{\left(\mathbf{x}_{i}-\mathbf{x}_{j}\right)}{\left|\mathbf{x}_{i}-\mathbf{x}_{j}\right|^{3}}=0$, $(i=1,\cdots,N_{inner})$ and $(j=1,\ldots,N_{surface})$,

where $N_{inner}$ and $N_{surface}$ are the numbers of inner and surface atoms, respectively. Thus, the resulting number of linear equations is $N_{inner}\times3$. Fig. S1 shows a spherical nanoparticle composed of 1,638 Cu atoms. Since having less number of interactions with neighboring atoms than inner atoms, the potential energy of the atoms close to the surface get increased in Fig. S1-a. The atoms of potential energy bigger than -350eV are identified as surface atoms (red) and the rest are inner atoms (blue).


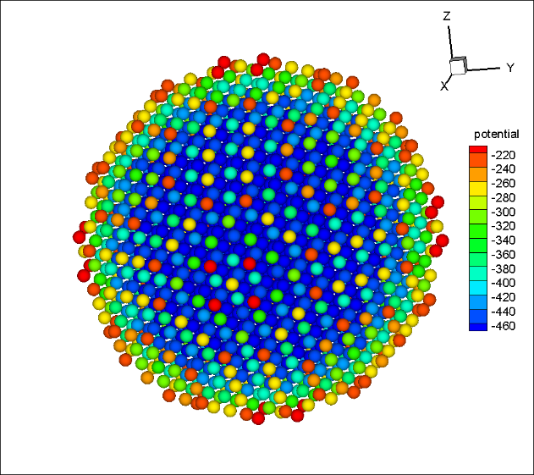

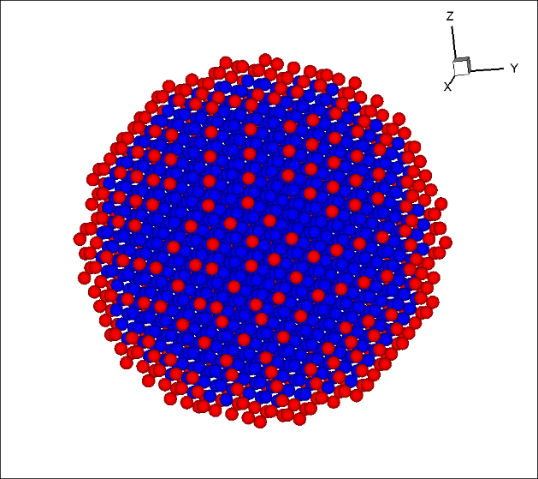


**Figure S1**. Interaction potential energy (a) and identified surface and inner atoms (b)

In the DEP process of AuNPs, the dipole moment of each atom is represented by $\mathbf{p}_{i}=\varepsilon\mathbf{E}$ and the electric field **E** by the gradient of electric potential ϕ as $\mathbf{E}=-\nabla\Phi$. The force at a position **x***_i_* of atom *i* is obtained by $\mathbf{f}_{i}=\varepsilon\mathbf{E}(\mathbf{x}_{i})\cdot\nabla\mathbf{E}(\mathbf{x}_{i})=\varepsilon\left(-\nabla\Phi\right)\cdot\nabla\left(-\nabla\Phi\right)$. If the electric field is spatially uniform, all the atoms are subjected to the same forces. Thus, for the change of morphology of AuNPs, the electric field should vary significantly. In the nanoscale region, since there are no significant changes in the magnitude and direction of the electric field, it is necessary to consider the redistribution of the charge in each atom that constitutes the AuNPs.

*Example*: MD simulations are performed to verify the interactions between AuNP-AuNP under a horizontally uniform electric field. The numerical models consist of 3 AuNPs forming a regular triangle and 4 AuNPs forming a square in Fig. S2. Each AuNP is a spherical nanoparticle of radius and the distance between two AuNPs is . An energy minimization of the MD system is conducted using a steepest descent algorithm. Furthermore, to remove the instability of the system, an isothermal (NVT) simulation is conducted for 100 ps at 300K with a time step of 2.0 fs. After thermal equilibrium, an electric field of is applied in the horizontal direction. The process of how ‘*pearl-chains*’ are formed can be seen. The dipole-dipole interaction causes the AuNPs to attract each other in the direction of the electric field, and repel each other in the direction perpendicular to the electric field. It turns out that the DEP force depends on the gradient of the electric field, while the AuNP-AuNP electrostatic forces depend on the intensity of the electric field. This implies that the more uniform the electric field is, the more important the AuNP-AuNP electrostatic force will be.


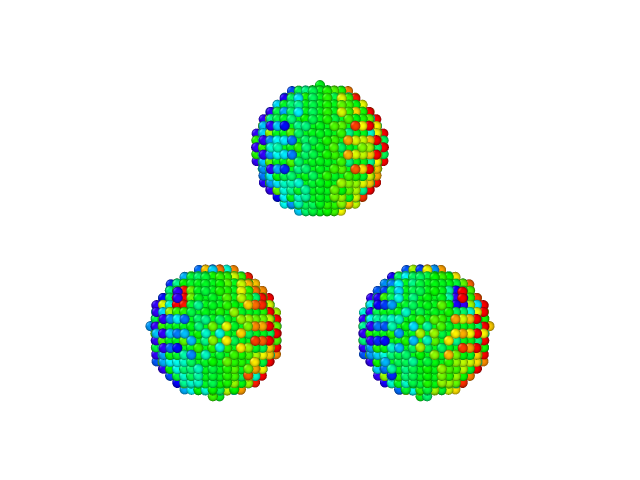

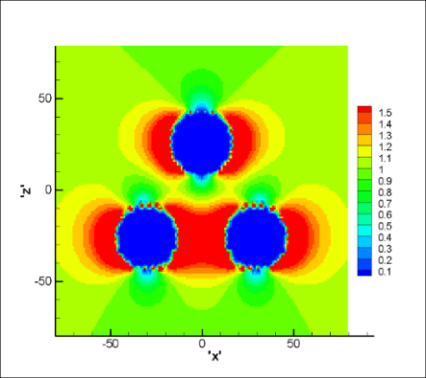

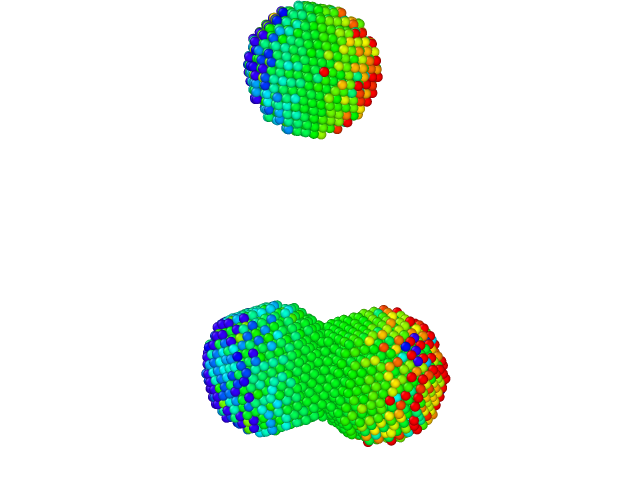

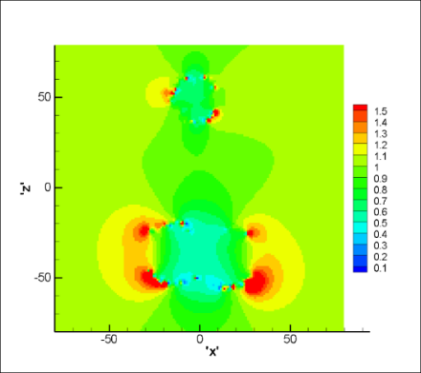


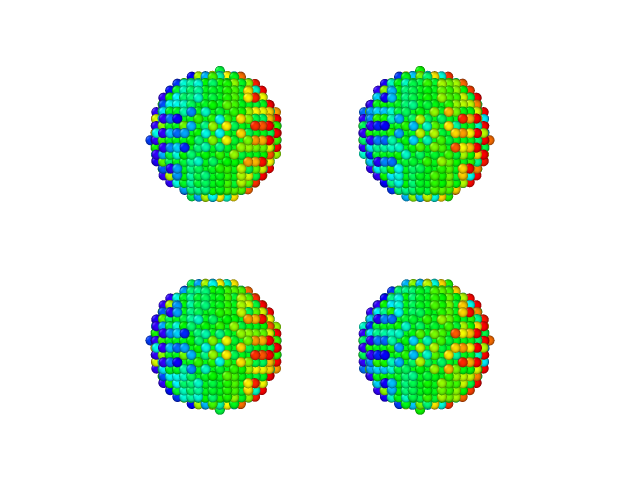

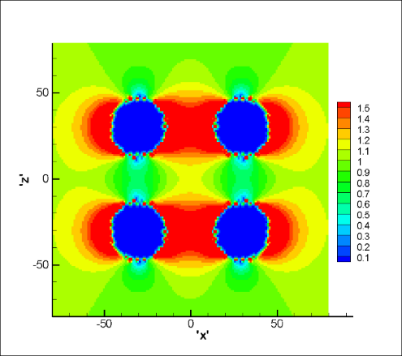

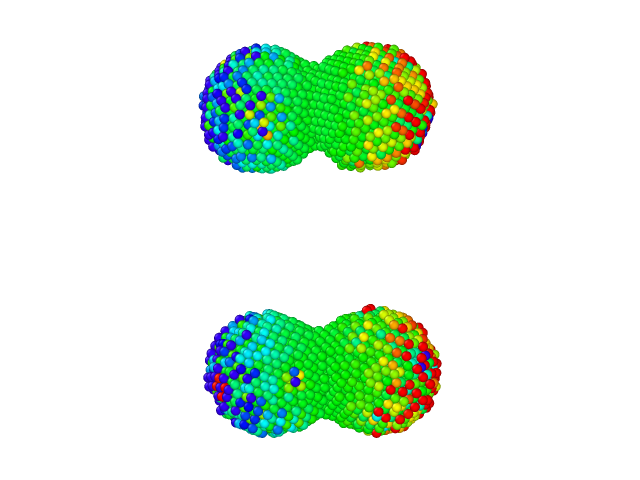

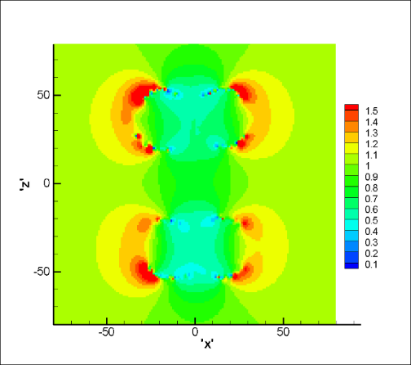


**Figure S2.** Charge distribution, Top: time evolution of 3 AuNPs from 0 ps to 175 ps,
bottom: time evolution of 4 AuNPs from 0 ps to 190 ps.

***2. Movie 1: motion of seven AuNPs on mica substrate (Refer to separate file)***

**Motion under electric field**: The process of how ‘*pearl-chains*’ are formed can be seen. The atoms located parallel to the direction of the electric field are charged with opposite polarity and attract each other. The dipole-dipole interaction causes the AuNPs to attract each other in the direction of the electric field. When two AuNPs are subjected to an electric field, an attractive force occurs due to the polarization of the AuNPs. One AuNP rotates in an opposite direction to the other one if the mica substrate is absent. In this case, however, all the AuNPs rotate in the same direction since the attraction force from the mica substrate is much bigger than that between the AuNPs. The rotation adjusting the lattice structure of each AuNP continues until cold welding occurs. After the aggregation, the charges in the aggregated AuNPs are redistributed. The aggregated AuNPs have the same polarity and thus repel each other in the direction perpendicular to the electric field.

***3. Movie 2: Evolution of centro-symmetry parameters for seven AuNPs (Refer to separate file)***

**Evolution of centro-symmetry parameter**: As the cold welding progresses, the stacking faults expand from the welding surface. After some relaxation period, the regular fcc structure is quickly recovered. The centro-symmetry analysis shows that the cold welding occurred with low stress, and at the end of the process, a crystalline structure with very few defects was achieved, recovering most of the original characteristics of the pristine AuNPs.
